# Veterinary Medical Association of New York County

**Published:** 1897-04

**Authors:** Robert W. Ellis

**Affiliations:** Secretary


### VETERINARY MEDICAL ASSOCIATION OF NEW YORK COUNTY.

THE meeting was called to order March 3d, at 8.30 P.M., at the Academy of Medicine, with the President, Dr. Huidekoper, in the chair. On roll-call the following members responded: Drs. Amling, Bretherton, C. C. Cattanach, J. S. Cattanach, Delaney, Ellis, Huidekoper, Hanson, Loomes, Machan, Miller, Murphy, Neher, O'Shea, Robertson, Ryder, and Winslow (17).

The minutes of the previous meeting were read and approved. Dr. Hanson read a carefully prepared paper on "Examination of Horses for Soundness," which was freely discussed by the members present. Moved and seconded that a vote of thanks be tendered Dr. Hanson for his paper; carried.

The Judiciary Committee (Dr. O'Shea, chairman) reported progress. Moved and seconded that the report be accepted; carried.

The Publication Committee, by President Huidekoper, reported progress. Moved and seconded that the report be accepted; carried.

An application for honorary membership was submitted as follows: "We hereby propose for honorary membership in the Veterinary Medical Association of New York County the name of Dr. W. Herbert Lowe, as a man whose every aim is for the advancement and elevation of the veterinary profession; taking a keen interest in veterinary and scientific associations in general, and an especial interest in this Association." Signed by Robert W. Ellis, H. D. Hanson, and J. E. Ryder. Referred to the Board of Censors, to report at the next meeting.

Dr. Neher reported a case of "Fatty Infiltration of the Heart in a Dog."

Additions to By-laws to be considered at the next meeting, to wit: Moved and seconded that in the discussion of papers each member shall be entitled to one hearing only, not to exceed five minutes, and that he shall confine himself to the subject, and the essayist shall make one final reply, closing the discussion.

Moved and seconded that the meeting adjourn; carried.

ROBERT W. ELLIS, D.V. S.,
Secretary.

---

The following resolutions were adopted by the New York State Medical Society at their last annual meeting:

It is the opinion of this Society that the State Board of Health should be permitted to make such regulations under the law as will provide for the proper inspection of suspected cattle and for the destruction of diseased animals, and especially that the services of a competent pathologist should be employed whenever such seem essential to the conduct of the service; but that we are not in favor of placing this important duty in the hands of a State Veterinarian except he be under the absolute control of the State Board of Health with reference to appointment, duties, and salary.

